# Antiproliferative Activity of Hinokitiol, a Tropolone Derivative, Is Mediated via the Inductions of p-JNK and p-PLCγ1 Signaling in PDGF-BB-Stimulated Vascular Smooth Muscle Cells

**DOI:** 10.3390/molecules20058198

**Published:** 2015-05-07

**Authors:** Po-Sheng Yang, Meng-Jiy Wang, Thanasekaran Jayakumar, Duen-Suey Chou, Ching-Ya Ko, Ming-Jen Hsu, Cheng-Ying Hsieh

**Affiliations:** 1 Department of Surgery, MacKay Memorial Hospital and Mackay Medical College, No. 92, Sec. 2, Zhongshan N. Rd., Taipei City 10449, Taiwan; E-Mail: psyangd0039@gmail.com; 2 Mackay Junior College of Medicine, Nursing, and Management, No. 92, Shengjing Rd., Beitou District, Taipei 112, Taiwan; 3 School of Nutrition and Health Sciences, Taipei Medical University, 250 Wu-Hsing St., Taipei 110, Taiwan; 4 Department of Pharmacology, School of Medicine, Taipei Medical University, 250 Wu-Hsing St., Taipei 110, Taiwan; E-Mails: mjwang@mail.ntust.edu.tw (M.-J.W.); tjaya_2002@yahoo.co.in (T.J.); fird@tmu.edu.tw (D.-S.C.); smallko1022@hotmail.com (C.-Y.K.); 5 Department of Chemical Engineering, National Taiwan University of Science and Technology, No. 43, Sec. 4, Keelung Rd., Da’an District, Taipei City 10607, Taiwan

**Keywords:** VSMC, hinokitiol, JNK1/2, PLC-γ1, PCNA, G0/G1

## Abstract

Abnormal proliferation of vascular smooth muscle cells (VSMCs) is important in the pathogenesis of vascular disorders such as atherosclerosis and restenosis. Hinokitiol, a tropolone derivative found in *Chamacyparis taiwanensis*, has been found to exhibit anticancer activity in a variety of cancers through inhibition of cell proliferation. In the present study, the possible anti-proliferative effect of hinokitiol was investigated on VSMCs. Our results showed that hinokitiol significantly attenuated the PDGF-BB-stimulated proliferation of VSMCs without cytotoxicity. Hinokitiol suppressed the expression of proliferating cell nuclear antigen (PCNA), a maker for cell cycle arrest, and caused G0/G1 phase arrest in cell cycle progression. To investigate the mechanism underlying the anti-proliferative effect of hinokitiol, we examined the effects of hinokitiol on phosphorylations of Akt, ERK1/2, p38 and JNK1/2. Phospholipase C (PLC)-γ1 phosphorylation, its phosphorylated substrates and p27^kip1^ expression was also analyzed. Pre-treatment of VSMCs with hinikitiol was found to significantly inhibit the PDGF-BB-induced phosphorylations of JNK1/2 and PLC-γ1, however no effects on Akt, ERK1/2, and p38. The up-regulation of p27^kip1^ was also observed in hinokitiol-treated VSMCs. Taken together, our results suggest that hinokitiol inhibits PDGF-BB-induced proliferation of VSMCs by inducing cell cycle arrest, suppressing JNK1/2 phosphorylation and PLC-γ1, and stimulating p27^kip1^ expression. These findings suggest that hinokitiol may be beneficial for the treatment of vascular-related disorders and diseases.

## 1. Introduction

Vascular smooth muscle cells (VSMCs) are associated in the development of numerous pathological conditions such as neointima formation, restenosis and atherosclerosis. VSMCs are normally in a quiescent state, presenting a well distinguished contractile phenotype, conversely, this phenotype would be lost after vascular injury, shifting to a synthetic phenotype providing an entry into the cell cycle and proliferation [1,2]. The dysfunctional VSMCs lead to abnormal proliferation, cell migration, expression of adhesion molecules, and modulation of the extracellular matrix, followed by the occurrence of pathogenic vascular complaints [3,4]. 

Platelet-derived growth factor (PDGF), produced by VSMCs has been considered to be important for the development of vascular diseases. PDGF-BB binds to the PDGF receptor (PDGF-R), and this then leads to phosphorylation on multiple tyrosine residues of PDGF-R followed by the stimulation of three major signal transduction pathways: the extracellular signal-regulated kinase (ERK)1/2, phospholipase C (PLC)-γ1, and phosphatidylinositol 3-kinase (PI3K)/Akt pathways [5,6]. Inhibitors of these signaling pathways have been shown to modulate neointimal formation in the balloon-injured arterial vessels of rats [7]. Cell cycle progression is controlled by enzymes such as cyclin E, cyclin A, cyclin B1, and proliferating cell nuclear antigen (PCNA); p27kip1 has been reported to inhibit the G0/G1 phases of the cell cycle by regulating some of these cell cycle related enzymes [8,9]. Therefore, the regulation of PDGF signaling pathway in VSMCs may be a good pharmacological approach for the prevention of atherosclerosis or vascular diseases.

**Figure 1:**
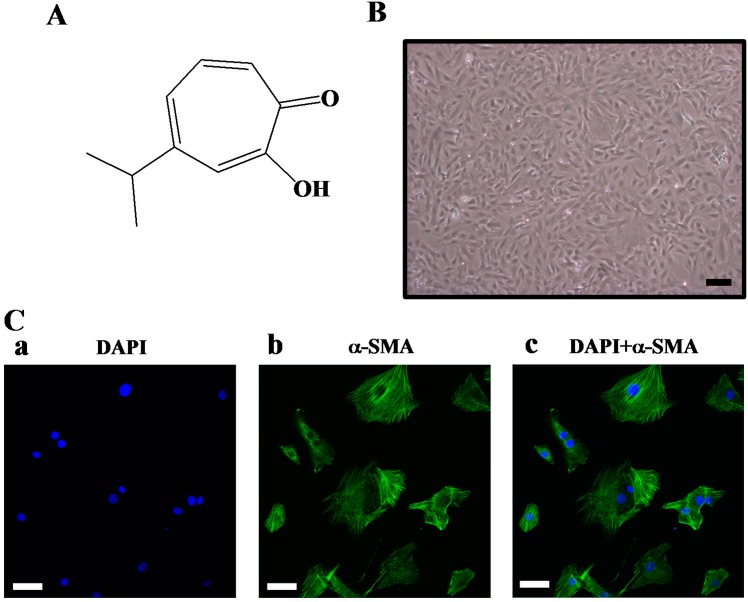
(**A**) The chemical structure of hinokitiol; (**B**) Cell morphology of primary cultured rat vascular smooth muscle cells. The black bar indicates 100 μm; (**C**) Representative confocal section of immunofluorescence staining for nuclear staining DAPI in blue and α-smooth muscle actin (SMA) in green. The white bar indicates 50 μm.

Hinokitiol (4-isopropyltropolone, Figure 1A) is a bioactive aromatic tropolone compound and a component of essential oils first isolated from the heart wood of *Chymacyparis taiwanensis*. It exerts antibacterial, antifungal, antiviral, and insecticidal activities [10]. This bioactive compound has been used in certain health care products, such as safe preservatives for toothpastes, cosmetics, hair tonics, mouth care gels, eyelid cleansers, hair restorers, skin lotions, and body soaps [11]. In our previous study, we found hinokitiol has potent antithrombotic effects in mice *in vivo* and antiplatelet activity *in vitro* [12]. Moreover, our recent studies also showed that hinokitiol exhibits neuroprotective [13] and *in vitro* and *in vivo* antitumor effects [14]. However, the effects of hinokitiol on VSMC proliferation have not yet been clarified. Therefore, this study aimed to explain the effects of hinokitiol on PDGF-BB-stimulated VSMC proliferation, as well as its mechanism of action in this context.

## 2. Results and Discussion

### 2.1. Results

#### 2.1.1. Effect of Hinokitiol on PDGF-BB-Induced Proliferation of VSMCs

**Figure 2:**
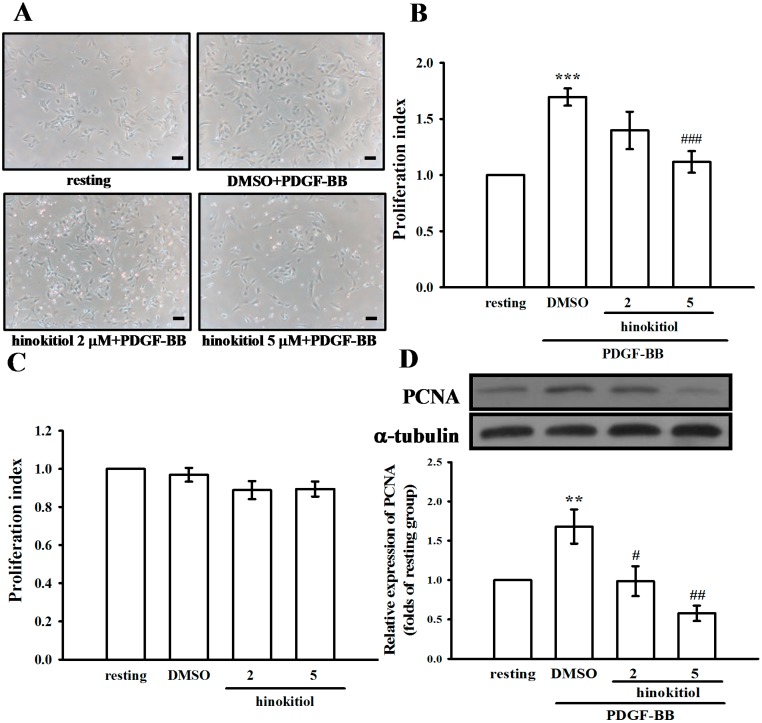
Effects of hinokitiol on the platelet-derived growth factor (PDGF)-BB-induced proliferation of vascular smooth muscle cells (VSMCs). (**A**) Morphological changes in VSMCs co-treated with PDGF-BB (10 ng/mL) and hinokitiol (2 and 5 µM) for 24 h were observed (scale bar = 100 µm); (**B**) VSMCs were co-treated with hinokitiol (2 and 5 µM) and PDGF-BB (10 ng/mL) for 24 h, and cell proliferation was determined using the MTT assay; (**C**) VSMCs were treated with hinokitiol (2 and 5 µM) alone for 24 h, and cell proliferation was determined using the MTT assay; (**D**) VSMCs were pre-trated in a serum-free medium in the presence or absence of hinokitiol (1–5 μM) for 24 h, and then stimulated with 10 ng/mL PDGF-BB for 24 h. VSMCs were stained with anti-PCNA antibody, and its protein expression levels were determined by immunoblotting analysis. Data are presented as the mean ± S.E.M. (A–C, *n* = 3; D, *n* = 4) ** *p* < 0.01 and *** *p* < 0.001 compared to the normal cells, ^#^ *p* < 0.05, ^##^ *p* < 0.01, and ^###^ *p* < 0.001, compared to the PDGF-BB stimulated cells.

To determine whether hinokitiol inhibits PDGF-BB-stimulated VSMC proliferation, we assessed the anti-proliferative effects of hinokitiol by cell morphological observation (Figure 2A). VSMCs were pre-incubated in the presence of hinokitiol (2 and 5 µM) in serum-free medium for 24 h and then stimulated with 10 ng/mL PDGF-BB for 24 h. Pretreatment with hinokitiol suppressed the PDGF-BB-stimulated cell numbers in a concentration-dependent manner (Figure 2B). Since anti-proliferative effect is often accompanied by cytotoxicity, we also measured the cytotoxic effects of hinokitiol on VSMCs, and found no evidence of cytotoxicity at 2 and 5 µM (Figure 2C). Thus, these results indicated that hinokitiol appeared to inhibit PDGF-BB-induced VSMCs proliferation without cytotoxicity.

#### 2.1.2. G0/G1 Phase Arrest of Hinokitiol in Cell Cycle Progression

We determined whether hinokitiol may modulate the cell cycle progression using flow cytometry analysis. VSMCs were stimulated with PDGF-BB along with the treatment of hinokitiol 2 and 5 µM. Hinokitiol increased the cell population of G0/G1 phase and decreased that of S phase. 

**Figure 3:**
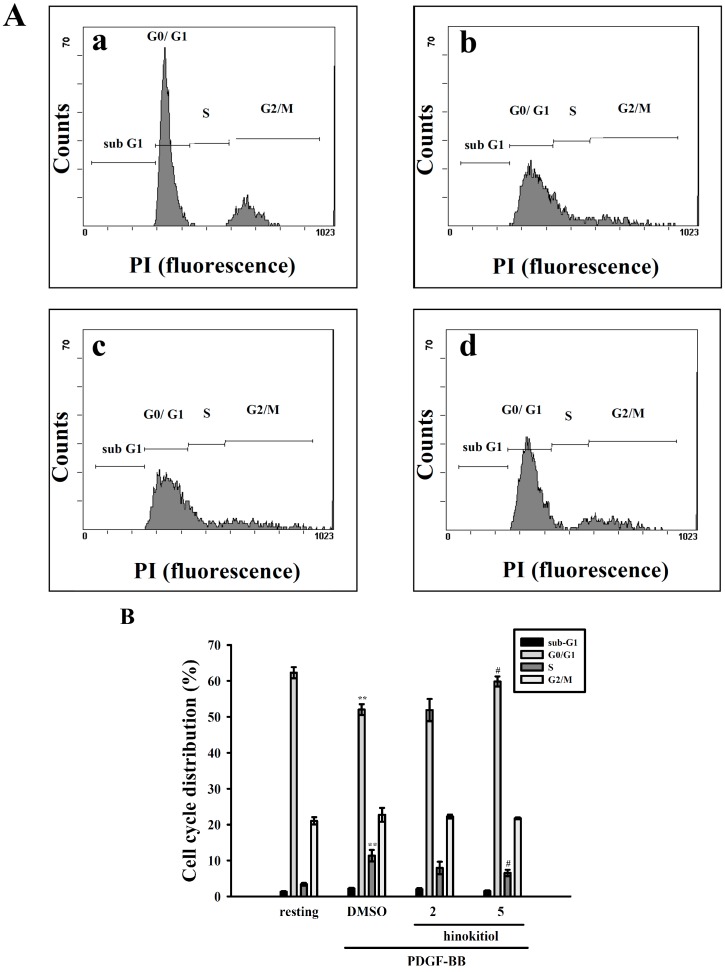
Effect of hinokitiol on PDGF-BB-induced cell cycle progression in VSMCs. (**A**) VSMCs were (graph **a**) treated with PBS (resting) or pretreated with (graph **b**) solvent control (0.1% DMSO) or (graph **c**) 2 μM or (graph **d**) 5 μM hinokitiol, followed by the addition of PDGF-BB (10 ng/mL) for 24 h. The cells were harvested by trypsinization. Then, the cells were fixed with 70% ethanol at 4 °C for 24 h, and then incubated at 4 °C overnight, after adding PI staining solution; (**B**) The percentages of cell population at each stage are expressed as mean values from four independent experiments. ** *p* < 0.01 compared to the normal cells, ^#^ *p* < 0.05 compared to the PDGF-BB stimulated cells.

PDGF-BB increased the S phase population of VSMCs; however, hinokitiol significantly reduced the cell population of S phase, indicating that hinokitiol may induce G0/G1 phase arrest (Figure 3A). On the other hand, we investigated the effect of hinokitiol on the levels of proliferating cell nuclear antigen (PCNA), as it involves the cell cycle progression from early G1 to S phase. PDGF-BB increased the expression of PCNA in VSMCs, whereas hinokitiol decreased its expression in a concentration-dependent manner (Figure 2D). These results suggest that hinokitiol may regulate the activities of PCNA, and then induce the G0/G1 cell cycle arrest in PDGF-BB-induced VSMCs (Figure 3B).

**Figure 4:**
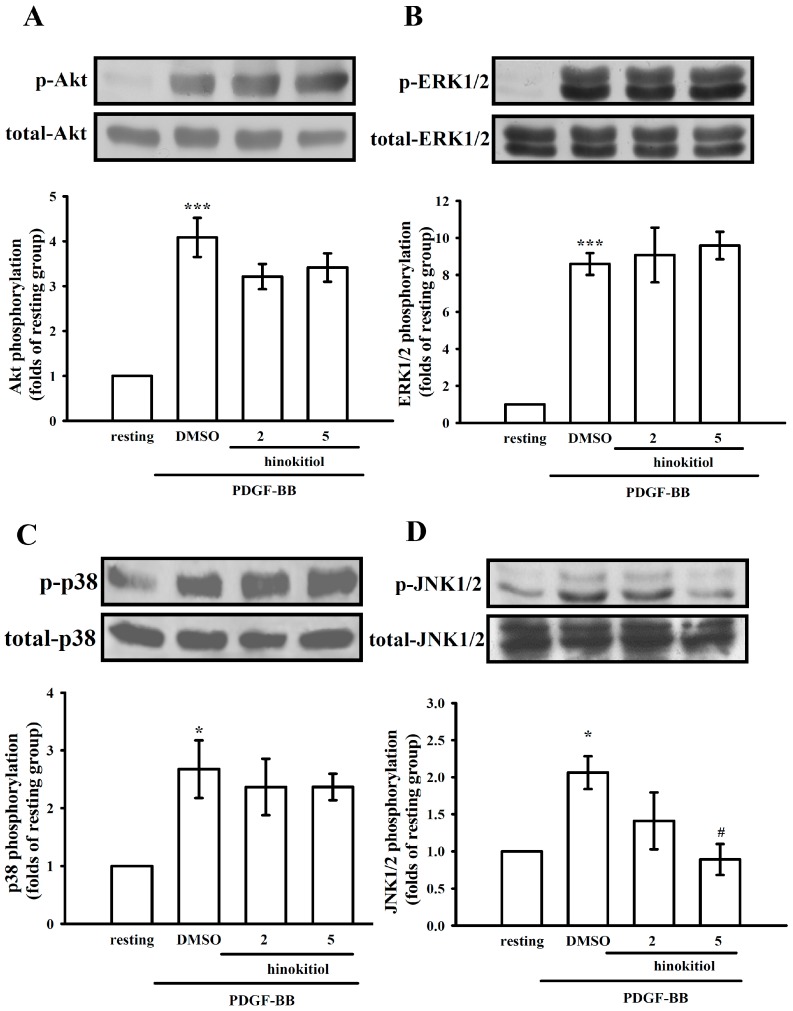
Effect of hinokitiol on PDGF-BB signaling pathway in VSMCs. Confluent cells were pre-treated in the presence or absence of hiokitiol (2–5 μM) in a serum-free medium, and then stimulated with 10 ng/mL PDGF-BB at 37 °C for 10 min. The cells were lysed, and the levels of (**A**) p-Akt; (**B**) p-ERK1/2; (**C**) p-p38 and (**D**) p-JNK were analyzed by SDS-PAGE and immunoblotting. The respective total proteins were used for the normalization of phosphorylated ones. Data are expressed as a mean ± S.E.M. (A–C, *n* = 3; D, *n* = 4) * *p* < 0.05, *** *p* < 0.001 compared to the normal cells, ^#^ *p* < 0.05 compared to the PDGF-BB stimulated cells.

#### 2.1.3. Down-Regulation of Hinokitiol in PDGF-BB Signaling Pathway

Activation of PDGF signaling pathway is associated with PDGF-BB-stimulated cell proliferation of VSMCs. For determining whether hinokitiol may inhibit the early signaling pathway of PDGF-BB, we measured the expression levels of p-Akt, p-ERK1/2, p-p38 and p-JNK1/2 in VSMCs treated with hinokitiol in the presence of PDGF-BB. Hinokitiol did not change the level of PDGF-BB-stimulated Akt, ERK, p38 phosphorylation (Figure 4A–C). However, only 5 μM hinokitiol in the presence of PDGF-BB showed the significant difference of p-JNK expression from the PDGF-BB alone-treated cells (Figure 4D). These results indicated that hinokitiol may inhibit cell proliferation through JNK signaling pathway.

#### 2.1.4. Hinokitiol on PDGF-BB Induced p-PLC-γ1, Its Substrate (Ser/Thr) Phosphorylation and p27^kip1^ Expression

**Figure 5:**
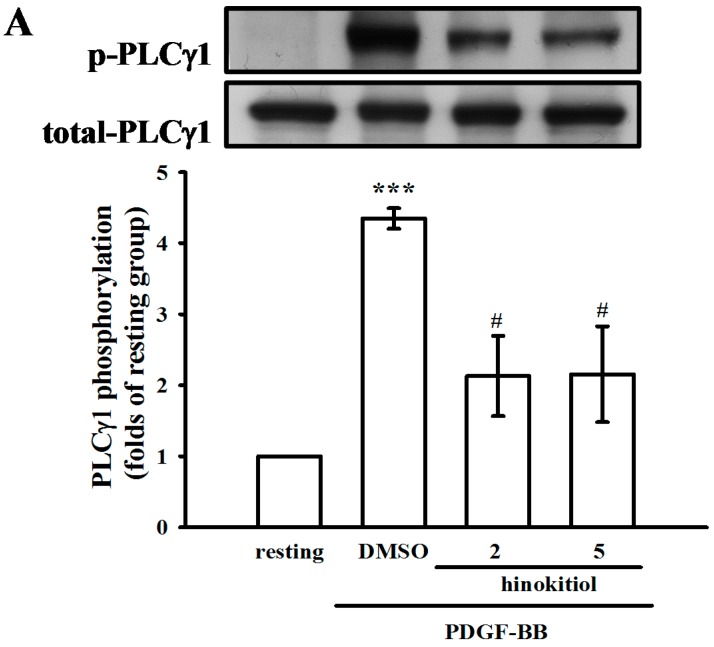

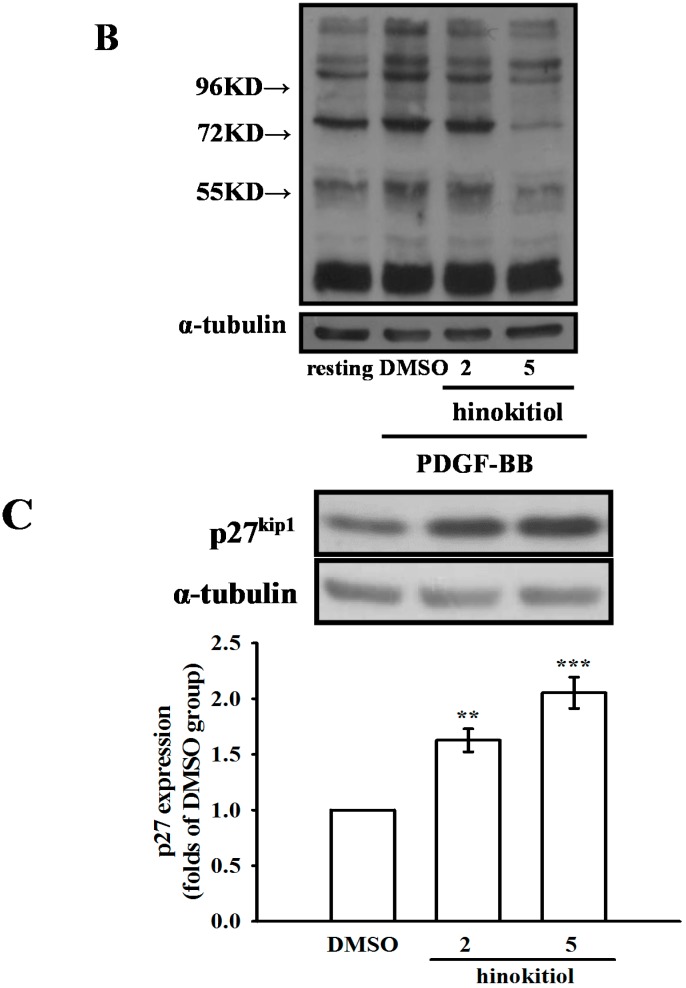
Effect of hinokitiol on PDGF-BB induced p-PLCγ1 and the expression of p27^kip1^ in VSMCs. (**A**,**B**) Confluent cells were pre-treated in the presence or absence of hiokitiol (2–5 μM) in a serum-free medium, and then stimulated with 10 ng/mL PDGF-BB at 37 °C for 10 min; (**C**) VSMCs which were cultured under normal condition were treated by hinokitiol (2–5 μM) for 24 h. The cells were lysed, and the level of p-PLCγ1, p-Ser/Thr substrates and p27^kip1^ were analyzed by SDS-PAGE and immunoblotting. The respective total protein was used for the normalization of phosphorylated ones. Data are expressed as a mean ± S.E.M. (A and C, *n* = 3; B, *n* = 4) ** *p* < 0.01 and *** *p* < 0.001 compared to the normal cells, ^#^ *p* < 0.05 compared to the PDGF-BB stimulated cells.

Previous studies have demonstrated that PLC-γl is activated by phosphorylation induced by receptor tyrosine kinases [15,16]. In order to examine this possibility, we detected the phosphorylation of PLC-γ1 and phospho-(Ser/Thr) substrate phosphorylation in PDGF-BB-induced VSMCs. PDGF-BB-induced PLC-γ1 phosphorylation was inhibited by hinokitiol in a concentration dependent manner (Figure 5A). Similarly, a phospho-(Ser/Thr) substrate Ab was used in this study to measure the phosphorylation of PKC protein targets. As shown in Figure 5B, Ser/Thr residues were phosphorylated upon PDGF-BB-induction in VSMCs. However, hinokitiol administration at 2 and 5 μM concentrations markedly inhibited these reactions. Furthermore, PLC-γ1 signaling pathway is known to be responsible for down-regulation of p27^kip1^, a cyclin dependent kinases (CDK) inhibitor- protein, in VSMC proliferation, and plays an important role in the pathology of neointimal formation [17]. As shown in Figure 5C, the treatment of hinokitiol (2 and 5 μM) significantly induced p27^kip1^ expression in VSMCs.

### 2.2. Discussion

Abnormal proliferation of vascular smooth muscle cells (VSMCs) induced by PDGF-BB- rises the risk of vascular disorders, and it acts as a critical contributing factor in the pathogenesis of atherosclerosis and restenosis [18,19], therefore, inhibition or modulation of VSMC proliferation is a major therapeutic strategy for atherosclerosis-related diseases [20,21]. In the present study, we examined the anti-proliferative activity of hinokitiol on VSMCs and the related signal transduction in cultured VSMCs. In this study, hinokitiol was presented to inhibit VSMCs proliferation without cytotoxicity. The anti-proliferative capacity of hinokitiol in VSMCs is correlated with induced G0/G1cell cycle arrest. The association between VSMCs proliferation and PCNA expression are serious events in the progression of atherosclerosis. PCNA, a protein synthesized early in the G1 and S phases of the cell cycle, functions in cell progression, DNA replication and DNA repair. PCNA acts as a sensitive marker of cell proliferation including VSMC proliferation [22]. Interestingly, we found that, PDGF-BB obviously increased VSMCs proliferation with augmented expression of PCNA. A previous study demonstrated that isorhynchophylline, an alkaloid from a traditional Chinese medicine, significantly blocked VSMCs progression by reducing the number of cells in S and G2/M phases and increasing cells in G0/G1 phase together with decreased expression of PCNA mRNA [23]. Therefore, the present study indicated that hinokitiol-mediated inhibition of PDGF-BB induced proliferation was related with a notable reduction in expression of PCNA in VSMCs. In addition, we also found that the treatment of hinokitiol (10 μM) could induce apoptosis in VSMCs, and this phenomenon was not observed in hinokitiol (2–5 μM)-treated VSMCs (supplemental Figure S1). It revealed that the reduction of VSMC numbers by hinokitiol maybe also due to increased cell apoptosis, especially treating with the high concentration of hinokitiol. However, the advantages of increased VSMC apoptosis in the treatment of atherosclerosis and restenosis are still controversial [24]. The dose of hinokitiol treatment is thus important for clinical application.

MAPKs are involved in cellular responses required for cell survival, including cell migration, proliferation, and differentiation [25]. In VSMCs, these responses are initiated when PDGF-BB induces the activation of downstream signaling molecules, such as by phosphorylation of ERK1/2 and p38 [26]. Our data show that hinokitiol reduces the phosphorylation of JNK1/2 inPDGF-BB-stimulated VSMCs in a dose-dependent manner, however it did not affect Akt, ERK1/2 and p38 phosphorylation. Among the three JNK protein kinases (JNK1, JNK2, and JNK3), JNK1 and 2 are universally expressed, and the expression of JNK3 is restricted to the brain, heart, and testes [27]. The activation of MAPK kinase kinases (MAPKKKs) by extracellular stimuli may consequently activate MAPK kinases 4 and 7 (MKK4 and MKK7), which phosphorylate JNKs. Stimulated JNKs result in the phosphorylation of many transcription factors, including the c-Jun component of the activator protein (AP)-1 transcription family [27,28]. It has been proposed that JNK knockdown can reduce cell migration and proliferation of PDGF-stimulated VSMCs [29,30]. In this study, hinokitiol inhibited JNK1/2 phosphorylation in VSMCs stimulated by PDGF-BB. Once PDGF-BB is bound to its receptor in smooth muscle cells, three major signal transduction pathways including p-PLCγ1, p-Akt, and p-ERK1/2 can be activated [31]. In this study, we found that hinokitiol had no effect on the expressions of p-Akt, p-ERK1/2 and p-p38 (Figure 4). Thus, the present results suggest that hinokitiol may inhibit the PDGF-BB-stimulated proliferation of VSMCs, through the blocking of JNK1/2 phosphorylation pathway followed by cell cycle arrest.

Three families of mammalian PLC isozymes, PLC-β, γ, and -δ, have been described based on their molecular structure and mechanism of regulation [32]. PLC-β isozymes have been shown to be activated by Gα and Gβγ subunits of the heterotrimeric G proteins, while PLC-γ isozymes are regulated by tyrosine phosphorylation [32]. PLCγ is the only isoform of PLC that becomes activated by tyrosine phosphorylation, leading to inositol trisphosphate production from intracellular stores [32]. Using a specific mAb, our studies demonstrate the induction of PLCγ1 tyrosine phosphorylation by PDGF-BB. Due to the concrete significance in terms of VSMC contraction and potential pharmacological applicability, our findings demonstrated that the PDGF-BB induced PLCγ1 phosphorylation is eliminated by hinokitiol. PLCγ1 was reported to play an important role in mitogenic responses in VSMCs, including cell migration and proliferation [33]. Activated PLCγ1 hydrolyzes its substrate, phosphatidylinositol 4,5-bisphosphate (PIP2), to produce two secondary messengers: IP3 and diacylglycerol. The former provokes the release of intracellularly stored Ca^2+^ to elevate cytoplasmic free Ca^2+^ levels, and the latter serves as an endogenous activator of protein kinase C (PKC) [34]. We thus investigated the influence of hinokitiol on PKC activation by measuring the phosphorylation of PKC target proteins (Ser/Thr substrates), and found that the treatment of hinokitiol significantly suppressed PDGF-BB-induced Ser/Thr substrate phosphorylation. It may further support that PLCγ1 activation was inhibited by hinokitiol in PDGF-BB-stimulated VSMCs. Furthermore, the treatment of U73122, a PLC inhibitor, was reported to attenuate JNK1/2 phosphorylation in mesenchymal stem cells and macrophages [35,36]. These studies might suggest that JNK1/2 is a downstream signaling molecule of PLC-γ1. On the other hand, p27^kip1^, a CDK inhibitor protein was known to play a key part in VSMC proliferation and neointimal formation [37]. JNK1/2 and PLCγ1 signaling pathways have been found to regulate cell proliferation by controlling the expression of p27^kip1^ in VSMCs [17,30]. In the present study, we also found the up-regulation of p27^kip1^ in hinokitiol-treated VSMCs. These evidences collectively indicate the role of p27^kip1^ in hinokitiol-reduced VSMC proliferation.

## 3. Experimental Section

### 3.1. Materials

Hinokitiol (product number: 469521; purity: ≥98.5%) and 3-(4,5-dimethylthiazol-2-yl)-2,5-diphenyl tetrazolium bromide (MTT) were purchased from Sigma Chemical Company (St. Louis, MO, USA). Recombinant PDGF-BB was purchased from PeproTech (Rocky Hill, NJ, USA). Anti-mouse and anti-rabbit immunoglobulin G-conjugated horseradish peroxidase (HRP) was purchased from GE Healthcare (Sunnyvale, CA, USA) and/or Jackson-Immuno Research (West Grove, PA, USA). The anti-p38 MAPK, anti-phospho-p42/p44 ERK (Thr202/Tyr204) and anti-phospho-c-Jun N-terminal kinase (JNK) (Thr183/Tyr185) mAbs, and anti-phospho-(Ser/Thr) substrate polyclonal antibody (pAb) were procured from Cell Signaling (Beverly, MA, USA). The anti-phospho-PLCγ1 (Tyr783) pAb was from Signalway Antibody (Pearland, TX, USA). The anti-p27^kip1^ mAb was purchased from Abcam (Cambridge, UK). The Hybond-P polyvinylidene difluoride (PVDF) membrane, and enhanced chemiluminescence (ECL) western blotting detection reagent and analysis system were obtained from GE Healthcare. All other chemicals used in this study were of reagent grade. Hinokitiol was dissolved in DMSO and stored at 4 °C until used.

### 3.2. Animal Care and Cultivation of Rat Primary VSMCs 

All animal experiments and care were performed according to the National Research Council Guide for the Care and Use of Laboratory Animals, and were approved by the Institutional Animal Care and Use Committee (IACUC) of Taipei Medical University. VSMCs were enzymatically isolated from the thoracic aortas of male Wistar rats (250–300 g) as previously described [37]. VSMCs were grown in Dulbecco’s modified Eagle’s medium (DMEM) supplemented with 20 mM HEPES, 10% fetal bovine serum (FBS), 1% penicillin/streptomycin, and 2 mM glutamine at 37 °C in a humidified atmosphere of 5% CO_2_. VSMCs at passage 4–8 were used in all experiments. Primary cultured rat aortic VSMCs showed a typical “hills and valleys” pattern when cells reached 90% confluence (Figure 1B), and the expression of α-smooth muscle actin was confirmed (Figure 1C). 

### 3.3. VSMC Viability Test and Proliferation Assay

VSMCs (2 × 10^4^ cells/well) were seeded on 24-well plates and cultured in DMEM containing 10% FBS for 24 h. The medium was then replaced with serum-free medium for 24 h. Serum starved VSMCs were pretreated with hinokitiol (1–5 μM), or 0.1% dimethyl sulfoxide (DMSO) for 20 min and then stimulated with PDGF-BB (10 ng/mL) for 24 h. The cell number was measured using a colorimetric assay based on the ability of mitochondria in viable cells to reduce the MTT as previously described [38]. The cell number index was calculated as the absorbance of treated cells/control cells × 100%. Cell morphology was observed and recorded using an inverted microscope (IX71; Olympus, Tokyo, Japan) equipped with a CCD camera.

### 3.4. Cell Cycle Progression Analysis 

The cell cycle was analyzed as previously described [37]. Briefly, starved VSMCs (2 × 10^5^ cells/dish) were pretreated with hinokitiol (1, 2 and 5 μM), or 0.1% DMSO for 20 min and then stimulated with PDGF-BB (10 ng/mL) for 24 h. After 24 h, cells were harvested by trypsinization, washed twice with PBS and fixed in 70% ethanol. Following two washes with PBS, fixed cells were incubated in RNase (50 μg/mL) for 30 min, followed by staining with propidium iodide (80 µg/mL) and Triton-X-100 (0.2%). Samples were incubated for 20 min and subjected to flow cytometric analysis.

### 3.5. Immunoblotting

Immunoblotting was performed as previously described [39]. Serum-starved VSMCs (2 × 10^5^ cells/dish) were treated with hinokitiol (1–5 μM) or 0.1% DMSO for 20 min, followed by the addition of PDGF-BB (10 ng/mL) for the indicated times. After treatment, proteins were extracted with lysis buffer. The lysates were centrifuged, the supernatant protein (50 μg) was collected and subjected to sodium dodecylsulfate polyacrylamide gel electrophoresis (SDS-PAGE), and the separated proteins were electrophoretically transferred onto 0.45-μm polyvinylidene difluoride (PVDF) membranes. The blots were blocked with TBST (10 mM Tris-base, 100 mM NaCl, and 0.01% Tween 20) containing 5% bovine serum albumin (BSA) for 1 h and were then probed with various primary antibodies. The membranes were incubated with HRP-linked anti-mouse IgG or anti-rabbit IgG (diluted 1:3000 in TBST) for 1 h. Immunoreactive bands were detected by an enhanced chemiluminescence (ECL) system. The bar graph depicts the ratios of quantitative results obtained by scanning reactive bands and quantifying the optical density using video densitometry (Bio-profil; Biolight Windows Application V2000.01; Vilber Lourmat, Marne La Vallée, France).

### 3.6. Confocal Microscopy

Confocal microscopy was used to identify primary cultured rat VSMCs. VSMCs (1 × 10^5^ cells/cover slip) were placed on cover slips and allowed to adhere in a cell culture incubator overnight. VSMCs were fixed with 4% paraformaldehyde for 30 min and permeabilized with 80% methanol for 15 min. After incubation with 3% skimmed milk in PBS for 60 min, the preparation was incubated overnight with a primary anti-α-smooth muscle actin Ab (1:80). Cells were then washed three times with PBS and exposed to the secondary Ab [FITC-conjugated anti-rabbit immunoglobin G (IgG) at 1:100, 1% BSA/PBS] for 2 h. The samples were counter-stained with DAPI and mounted with mounting buffer (Vector Laboratories, Burlingame, CA, USA) under a glass cover slip on a Leica TCS SP5 Confocal Spectral Microscope Imaging System using an argon/krypton laser (Mannheim, Germany).

### 3.7. Statistical Analysis

The experimental results are expressed as mean ± S.E.M. One-way analysis of variance (ANOVA) was used for multiple comparisons (Sigma Stat v3.5 software, Systat Software Inc., San Jose, CA, USA). If there was a significant variation between treated groups, the Student-Newman-Keuls test was applied. A *p* value < 0.05 was considered to be statistically significant.

## 4. Conclusions

**Figure 6:**
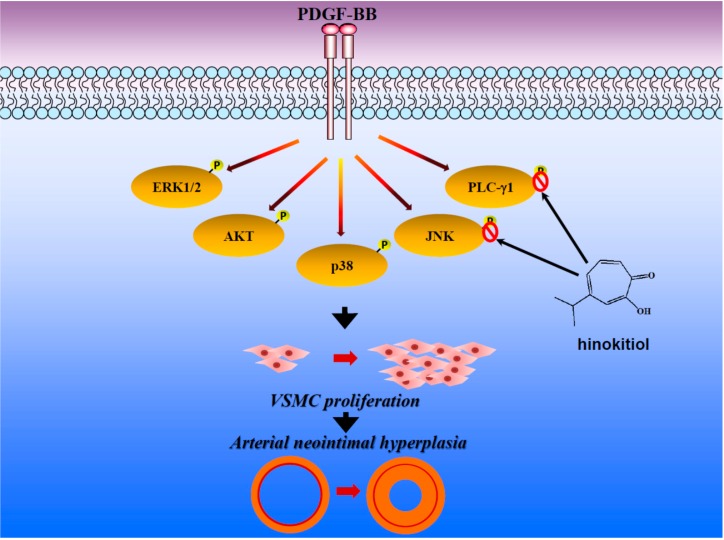
Hypothetical scheme of the inhibitory mechanism of hinokitiol in VSMC proliferation stimulated by PDGF-BB.

In conclusion, our results demonstrated that hinokitiol, a tropolone derivative, inhibits VSMC proliferation via JNK1/2 and PLC-γ1 phosphorylation (Figure 6) and limiting the synthesis of specific cell cycle enzyme, PCNA, which in turn prevents the abnormal proliferation of VSMCs by inducing cell cycle arrest at the G0/G1 phase. Though our experiments with cultured vascular smooth muscle cells may not completely described the actual mechanism of hinokitiol in VSMCs, the obtained results may offer a new insight into the anti-cardiovascular agents of hinokitiol.

## Supplementary Materials

Supplementary materials can be accessed at: http://www.mdpi.com/1420-3049/20/05/8198/s1.
