# Supplementary material for: Antiproliferative Activity of Hinokitiol, a Tropolone Derivative, Is Mediated via the Inductions of p-JNK and p-PLCγ1 Signaling in PDGF-BB-Stimulated Vascular Smooth Muscle Cells
Source: Molecules. 2015 May 7;20(5):8198–212. doi: 10.3390/molecules20058198 (PMC6272725; doi:10.3390/molecules20058198)
Supplement: Supplementary file 1 [file molecules-20-08198-s001.pdf]

Supplementary Materials

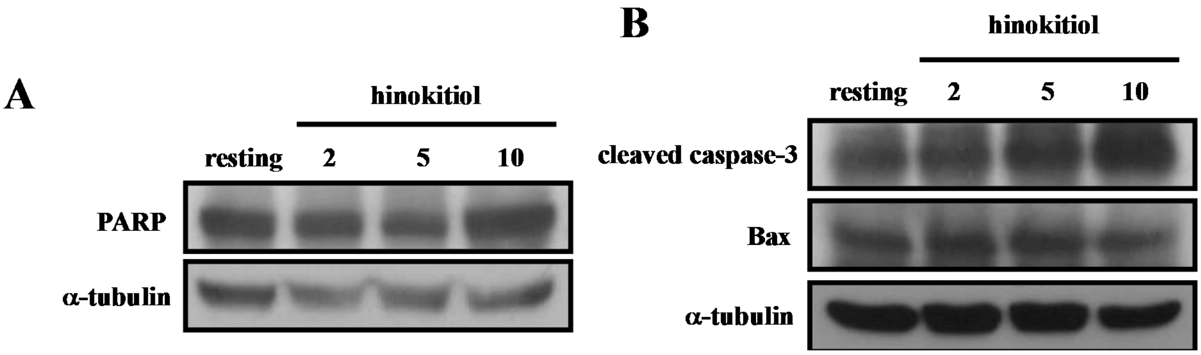

Figure S1. Effect of hinokitiol on PARP, cleaved caspase-3, and Bax expression in VSMCs. VSMCs were pre-treated in the presence or absence of hiokitiol (2–10 μM) for 24 h, and the cells were then lysed. The level of (**A**) PARP, (**B**) cleaved caspase-3, and Bax was analyzed by SDS-PAGE and immunoblotting. Data are representative of two separated experiments with similar results.

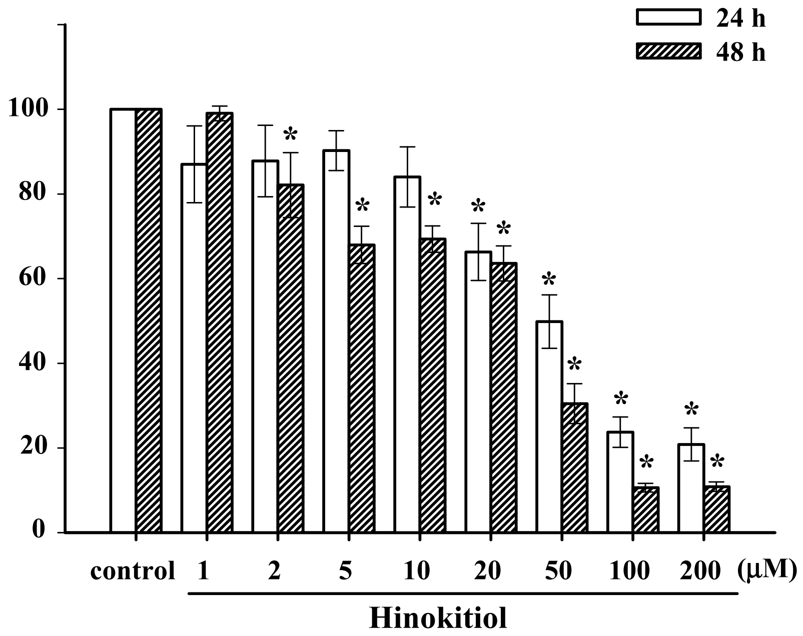

Figure S2. Effects of hinokitiol on cell proliferation of vascular smooth muscle cells (VSMCs). VSMCs were co-treated with hinokitiol (1–200 μM) for 24 or 48 h, and cell proliferation was determined using the MTT assay. Data are presented as the mean ± S.E.M. (n = 3). * $p < 0.05$ compared to the control group.
